# Role of community-based measures in adherence to self-protective behaviors during first wave of COVID-19 pandemic in Saudi Arabia

**DOI:** 10.34172/hpp.2021.10

**Published:** 2021-02-07

**Authors:** Asma Ayyed AL-Shammary, Sehar un-Nisa Hassan, Aqeela Zahra, Fahad Bin Zafir Algahtani, Shadi Suleiman

**Affiliations:** ^1^College of Public Health and Health Informatics, College of Science, University of Ha’il, Ha’il-81451, Kingdom of Saudi Arabia; ^2^Department of Public Health, College of Public health and Health Informatics, University of Ha’il, Ha’il-81451, Kingdom of Saudi Arabia; ^3^Department of Family and Community Medicine, College of Medicine, University of Ha’il, Ha’il-81451, Kingdom of Saudi Arabia; ^4^Molecular diagnostic & Personalized Therapeutic Unit, University of Ha’il, Ha’il-81451, Kingdom of Saudi Arabia; ^5^College of Applied Medical Science, University of Ha’il, Ha’il-81451, Kingdom of Saudi Arabia

**Keywords:** COVID-19 pandemic, Community health, Health behaviors, Prevention and control, Preventive measures, Public health, interventions

## Abstract

**Background:** The expected second wave of the COVID-19 pandemic has started in various regions of the world. Public health experts warned that it could be as lethal as the first wave if people did not comply with self-protective measures. Currently, there is a gap in the literature on the relationship between peoples’ assessment of the effectiveness of community-based measures regarding adherence to self-protective behaviors for COVID-19 prevention and control. This study aimed to assess the role of the perceived effectiveness of community-based measures in adherence to self-protective behaviors during the COVID-19 pandemic.

**Methods:** The cross-sectional online survey conducted from March 24 to June 22, 2020. The study sample Included 400 participants (49% male and 51% female) from the Kingdom of Saudi Arabia (KSA). The outcome measure was compliance to four self-protective behaviors i.e., "social distancing;" "wearing facemask;" "washing hands more frequently;" and "disinfecting surfaces in homes." We computed Chi-square statistics and odds ratios (ORs) using 95% confidence intervals (CIs).

**Results:** The findings demonstrated that participants aged 25–34 years old were 25% less likely to comply with hand hygiene (OR = 0.75; 95% CI: 0.33–0.95) and social distancing (OR = 0.76; 95% CI: 0.34–0.98). Misconceptions related to COVID-19 significantly decreased compliance with self-protective behaviors by up to 27%. Participants who rated government decisions as useful were approximately 1.7 times more likely to comply with self-protective behaviors.

**Conclusion:** Community-based measures should focus on engaging segments of the population That are currently less compliant. Health education policies should also focus on enhancing the perceived sense of control and personal responsibility and reduce anxiety levels. A continuous commitment to the implementation of preventive interventions and the clarification of misconceptions are required to combat the expected second wave.

## Introduction

In December 2019, a cluster of atypical pneumonia cases was reported in Wuhan city, China. The cause was unknown; however, it became evident that a novel coronavirus strain could pass quickly through human-to-human interaction, and the virus could survive on surfaces for a relatively longer duration. Due to its highly infectious nature, on January 2020, the World Health Organization (WHO) warned that the global pandemic is associated with a high fatality ratio, thus requiring quick communication and coordination to implement mitigation measures. The immediate preventive measures recommended by WHO and other health organizations were to quarantine the infected individuals, maintain social distance, hand hygiene, and use facemasks.^1^ Given the lack of progress in the creation of vaccines against the novel coronavirus, WHO guidelines were adopted and enforced by the governments in several countries around the world. These included tracing, treating, and isolating people who have the infection, curfews, and the implementation of longer lockdowns, as well as educating the public to adopt preventive measures. The Saudi government also took several community-based measures to prevent the spread of coronavirus disease (COVID-19) during the first pandemic phase. These included the provision of facemasks, hand gloves, and hand sanitizers in all workplaces/markets, cleaning and disinfecting public places, community surveillance to prevent the movement and gathering of people, closure of mosques/community halls, and hefty fines for those who did not comply with wearing facemasks and social distancing. The implementation of these measures created a new community environment, which is likely to impact the public’s health behaviors. In the context of the COVID-19 pandemic, other than implementing community-based measures, reports on the incidence and fatality ratio shown on electronic and social media 24/7 also influenced people’s psychosocial responses.

Literature shows that psychological responses during the pandemic are essential determinants of compliance with self-protective measures.^2^ Fear responses and risk perceptions have a significant influence on behavioral change.^3,4^ Health education and media campaigns use various methods to motivate the public to comply with self-protective measures. The assessment of psycho-behavioral responses of people during the severe acute respiratory syndrome (SARS) outbreak suggests that people often respond better to official public service announcements.^5^ Perceptions about disease severity/fatality, perceived effectiveness, and outcomes of recommended actions have shown effectiveness in determining compliance with personal self-protective behaviors during the SARS outbreak.^6,7^ Literature demonstrates that shallow risk perceptions can lead to less compliance with protective measures during viral outbreaks in the community.^8^ Previous studies point towards the negative role of uncertainty in the adoption of precautionary behaviors in the time of epidemics.^9,10^

The quick spread of the disease over a short period of time, lack of effective treatments, and high fatality ratio were the most relevant factors in determining people’s risk perceptions and psycho-behavioral responses during the COVID-19 pandemic.^2,3^ Recent studies from China validate a positive impact of government interventions on the public’s adoption of precautionary measures and its relationship with the prevention and control of COVID-19.^11,12^ Some previous studies indicate the role of public cooperation in achieving positive outcomes in the crisis response and community interventions implemented by the governments.^13^ It explains that perceptions of threatening events motivate people to adopt self-protective behaviors. Furthermore, beliefs in performing preventive behaviors can effectively reduce the threat and also have a significant role in compliance with preventive actions. Perceived effectiveness in relation to community-based measures is the “subjective probability” that a public health campaign will have a convincing impact. These perceptions about community-based measures’ effectiveness may have a significant role in community engagement for infection control during epidemics.^14^

Therefore, it is crucial to study how community-based measures relate to the public’s adherence to self-protective behaviors at an individual level. The COVID-19 pandemic is unique in many respects. There are still gaps in the investigation of the relationship between people’s beliefs about the usefulness and effectiveness of community measures regarding compliance with precautionary measures across different social and economic contexts of countries and regions. Most of the available literature is from China and other Western countries, and there is a paucity of evidence from the Middle East region. This study aimed to fill these gaps by focusing on three contributions from this analysis. Firstly, we assessed the fear/anxiety levels, risk perceptions, common misconceptions related to COVID-19, public perceptions about the usefulness/effectiveness of community measures implemented, and adherence to self-protective behaviors. Secondly, we determined the nature of the relationships among disease incidence, fear levels, and risk perceptions during the first wave of the pandemic. Thirdly, we examined the predictive role of fear/anxiety levels, risk perceptions, misconceptions related to COVID-19, and ratings of the role of community-based measures in compliance with self-protective behaviors during the first wave of the pandemic in the Kingdom of Saudi Arabia (KSA).

## Materials and Methods

### Study design and procedure

We conducted a cross-sectional online survey from March 24 to June 22, 2020, by making a survey link available for one week each month. It was an appropriate strategy to gather representative sub-sets of survey data over a period of four months in order to achieve more variance along with precision and accuracy in estimations.^15^ According to statistical estimations, the minimum sample size was (n=385), whereas the assumption of the proportion of adherence to self-protection behaviors toward COVID-19 is 50%, with a 95% confidence interval (CI) and 0.05 precision.^16^ We recruited the study participants by sending an online study invitation link to target populations through other professional colleagues residing in various regions of KSA. In order to maximize the reach, the study invitation link was shared through various social media platforms. After providing their informed consent, the participants completed the survey questionnaire. The response rate was approximately 74%.

### Survey questionnaire

We designed the survey questionnaire based on an extensive review of previous literature and previous survey questionnaires that assessed similar constructs.^17-20^ The items included in the study questionnaire were constructed following the BRUSO model, which focuses on the following characteristics of items: ”brief,” “relevant,” “unambiguous,” “specific,” and “objective.”^21^ This model has been found to be effective in minimizing unintended context effects and maximizing the reliability and validity of participants’ responses.

### Demographic information and travel history

In this section, we collected data on demographic variables including gender, age, highest educational level, profession, marital status, number of children, current place of residence in Saudi Arabia, and travel history.

### Psychological factors

This includes assessing the participants’ fear/anxiety levels related to COVID-19 and risk perceptions related to COVID-19 infection. A 10-point rating scale was used to obtain participants’ ratings of their fear/anxiety levels and perceptions of their risk of catching the COVID-19 virus, with a score of 1 being the lowest and a score of 10 being the highest.

### Misconceptions related to COVID-19

A questionnaire comprising eight items assessed the prevalence of various misconceptions related to the COVID-19 pandemic. The sample item was “*New coronavirus infection only affects older and already sick people.”* The response category for each item was either yes = 1 or no = 0. The total score ranged from 0 to 8, with a score of 0 indicating the lowest level and 8 indicating the highest level.

### Perceived effectiveness of community-based measures

The Saudi government implemented several measures to prevent the spread of COVID-19 in the community. These include the provision of facemasks, hand gloves, and hand sanitizers in all workplaces and marketplaces, the implementation of standard procedures to clean and disinfect public places, community surveillance to prevent gathering of people for social activities, closure of mosques, community hall, etc. There were legal implications in the form of heavy fines set in order to control the non-compliant behavior of community residents. A set of five questions obtained participants’ ratings on the effectiveness of preventive measures implemented in marketplaces, workplaces, healthcare organizations, travel places, and effectiveness of government decisions. The sample item was *“How would you rate the effectiveness of decisions taken by the governmental authorities to prevent the spread of the new coronavirus in the community?*”Participants rated the effectiveness of the community measures and government decisions on a 10-point rating scale, with a score of 1 indicating “Useless” and a score of 10 indicating “Very Useful.”

### Adherence to self-protective behaviors

According to WHO guidelines on COVID-19 infection control, people should adhere to four self-protective behaviors: “wearing facemask,” “hand hygiene,” “social distancing,” and “disinfecting surfaces.” Participants were asked to report their compliance to these four self-protective actions. The response categories included “No = 0” and “Yes always = 1.”

### Weekly case-incidence

Data on the weekly case incidence during months of data collection were obtained from the Saudi Ministry of Health resources in order to inspect the pattern of association between case incidence, fear/anxiety levels, and risk perceptions.

### Statistical analysis

IBM Statistical Program for Social Sciences (SPSS) version 20 was used for statistical analysis.^22^ Results are presented as frequency (%) for categorical variables and mean (SD) for continuous variables. In this study, adherence to self-protective behaviors was considered a binary outcome (No/Yes Always). The association of demographic variables, psychological factors, misconceptions related to COVID-19, and perceived effectiveness of community-based measures with adherence to self-protective behaviors was evaluated using the chi-square test and *t* test. Logistic regression models were used to determine the role of perceived effectiveness of community-based measures in the adoption of self-protective behaviors after adjusting for demographic variables, psychological factors, and misconceptions related to COVID-19. The findings are presented as odds ratio (OR) with 95% CI choosing p-value significance at *P* < 0.001, *P* < 0.01, and *P* < 0.05 for all predictor variables. A line graph was developed in Excel to present a temporal comparison of new cases of COVID-19, fear/anxiety levels, and risk perceptions. The missing data on the study variables were significantly reduced by using the option of compulsory response in this online survey questionnaire. The red asterisk suggests the respondents to provide answers to any missed questions at the time of submission of the form and accepts only complete forms.

## Results

### Demographic characteristics of respondents

The demographic characteristics of the participants are presented in Table 1. A total of (N=400) participants living in Saudi Arabia completed these online surveys distributed during the first wave of the COVID-19 pandemic period between March 24 and June 22, 2020. The sample comprised men (49%) and women (51%). The mean age of respondents was 37.6 years (SD = 10.8). Most participants (70%) were married, and 30% had more than four children. Regarding education, 63% had university education, 23% had college education, and 14% had up to secondary-level education. Regarding profession, 22% were in administrative and government jobs, 21% were housewives, 20% were teachers, and 16% were in healthcare. Regarding travel history, 67% of the participants reported not having travelled in the past two months (Table 1).

### Adherence to four self-protective behaviors across the demographic profiles

Table 1 shows the bivariate association of demographic variables with their adherence to four self-protective behaviors (wearing facemasks, hand hygiene, social distancing, and disinfecting surfaces). The findings showed that a higher percentage of female participants reported strict adherence to social distancing (*P* < 0.01) and sanitizing surfaces (*P* < 0.05). A higher percentage of participants with college education reported adherence to wearing facemasks (*P* < 0.05) and sanitizing surfaces (*P*< 0.05). A higher percentage of participants working in business reported compliance with wearing facemasks (*P* < 0.01), hand hygiene (*P* < 0.05), and sanitizing surfaces (*P* < 0.001). A higher percentage of married participants reported strict adherence to facemasks (*P* < 0.05) and social distancing (*P* < 0.01). A higher percentage of participants who had more than four children complied with wearing facemasks (*P* < 0.01) and social distancing (*P* < 0.01). Participants who traveled in the past two months were more likely to adhere to wearing facemasks (*P* < 0.05), hand hygiene (*P* < 0.05), and disinfecting surfaces (*P* < 0.05).

### Incidence of new cases of COVID-19, fear/anxiety levels, and risk perceptions during the first wave of the pandemic in KSA

**Figure 1:**
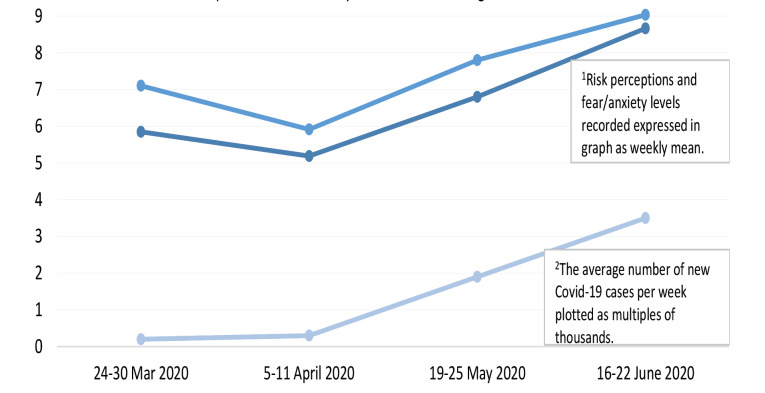
Temporal comparison on new cases of COVID-19, fear levels and risk perceptions during four weeks of data collection across four months (March- June 2020)

Figure 1 presents a temporal comparison of new cases of COVID-19, fear/anxiety levels, and risk perceptions during the four weeks of data collection spread across four months (March 24 to June 22, 2020), which is the total duration of this study. There was an increasing temporal trend in the mean fear/anxiety levels and risk perceptions, demonstrating a positive linear association with case incidence during these observation weeks. The correlation analysis showed that there was a strong positive statistically significant association between fear/anxiety levels and risk perceptions (r=0.72; *P* < 0.001).

### Association of predictor variables with adherence to self-protective behaviors

The comparison of mean scores of participants who adopted self-protective behaviors and those who did not, across psychological variables, misconceptions, and community-based measures, are presented in Table 2. The analysis demonstrates that fear/anxiety levels and risk perceptions are significantly associated with three self-protective behaviors: wearing a facemask and disinfecting surfaces (*P*< 0.001), and “social distancing” (*P* < 0.01). Participants who had higher risk perceptions were also more likely to comply with hand hygiene (*P* < 0.01) (Table 2). Participants who did not comply with precautionary behaviors, including wearing facemasks, social distancing, and disinfecting surfaces, had significantly higher mean scores on misconceptions related to COVID-19 (*P* < 0.001). In Table 2, the findings show that participants who perceived higher effectiveness of preventive measures implemented in workplaces and higher effectiveness of government decisions were more likely to comply with wearing facemasks (*P* < 0.001) and hand hygiene (*P* < 0.001). This relationship was also significant for hand hygiene (*P* < 0.05). The findings demonstrate that participants who perceived higher effectiveness of community measures implemented in marketplaces, workplaces, healthcare organizations, travel places, and higher effectiveness of government decisions were more likely to comply with social distancing and disinfecting surfaces either at *P* < 0.001, *P* < 0.01, or *P* < 0.05 (Table 2).

Table 3 shows the odds ratios (95% CI) and *P* values to demonstrate the predictive role of demographic variables, psychological variables, misconceptions, and perceived effectiveness of community-based measures with regard to the adoption of the four self-protective behaviors (wearing a facemask, hand hygiene, social distancing, and disinfecting surfaces) during the first wave of the COVID-19 pandemic in KSA.

### Predictors of wearing facemask

Among demographic factors, participants who attained a bachelor’s degree (OR = 2.02; 95% CI: 1.13–3.61), participants who were in business by profession (OR = 2.74; 95% CI: 1.03–7.28), and participants who were married (OR = 1.55; 95% CI: 0.98–2.44) were more likely to adhere to wearing a facemask. Among psychological factors, fear scores and risk perceptions increased compliance with wearing facemasks up to 1.2 times (OR = 1.23; 95% CI: 1.14–1.33 and OR = 1.21; 95% CI: 1.13–1.30, respectively). Misconceptions related to COVID-19 significantly decreased the likelihood of compliance with wearing facemasks by up to 27% (OR = 0.78; 95% CI: 0.72–0.85). With regard to the perceived effectiveness of community-based measures, participants who reported higher ratings in the effectiveness of measures implemented in healthcare settings had significantly increased likelihood of wearing facemasks (OR = 1.16; 95% CI: 1.04–1.28), and participants who rated government decisions as effective were also more likely to adopt the precautionary measure of wearing facemasks(OR = 1.42; 95% CI: 1.19–1.68).

### Predictors of hand hygiene

Participants in the younger age group (25–34 years) were 25% less likely to comply with hand hygiene (OR = 0.75; 95% CI: 0.33–0.95). Participants who traveled in the past two months were 45% more likely to comply with hand hygiene (OR = 2.45; 95% CI: 0.99–6.07). Among psychological factors, risk perceptions significantly increased compliance with hand hygiene by up to 13% (OR = 1.13; 95% CI: 1.01–1.27). Having misconceptions related to COVID-19 significantly decreased the likelihood of compliance with hand hygiene by up to 14% (OR = 0.86; 95% CI: 0.77–0.98). Participants who rated government decisions as effective were 33% more likely to adopt the precautionary measure of hand hygiene (OR = 1.33; 95% CI: 1.08–1.63).

### Predictors of social distancing

Findings demonstrated that female participants were two times more likely to comply with social distancing (OR = 2.18; 95% CI: 1.15–4.13) than male participants.Participants in the younger age group (25–34 years) were 24% less likely to comply with hand hygiene (OR = 0.76; 95% CI: 0.34–0.98). Housewives were 3.6 times (OR = 3.61: 95% CI: 1.25–10.36) more likely to comply with social distancing than those in administrative and government jobs. Participants who were married were 2.2 times (OR = 2.22; 95% CI: 1.21–4.13) more likely to adhere to social distancing precaution compared to those who were not married, while those who had more than four children were more 3.4 times (OR = 3.34; 95% CI: 1.37–8.15) more likely to adhere to social distancing compared to those who had no children. Both fear scores (OR = 1.14; 95% CI: 1.03–1.27) and risk perceptions (OR = 1.16; 95% CI: 1.05–1.28) significantly increased compliance with social distancing by up to 1.1 times. Having misconceptions related to COVID-19 significantly decreased the likelihood of adherence to social distancing by 14% (OR = 0.86; 95% CI: 0.78–0.96). Participants who rated the effectiveness of community measures implemented in marketplaces, workplaces, healthcare settings, and travel places had significantly increased chances of adherence to social distancing by up to 13–26%.

### Predictors of disinfecting surfaces

Among the demographic variables, housewives were 4.2 times more likely to adhere to the precautionary behavior of disinfecting surfaces (OR = 4.21; 95% CI: 1.71–10.3). Additionally, participants who were in business were five times more likely to regularly adhere to disinfecting surfaces (OR = 5.08; 95% CI: 1.65–15.6). Participants who traveled in the past two months were also more likely to abide by this precautionary behavior (OR = 1.71; 95% CI: 1.06–2.77). Regarding psychological factors, both fear scores and risk perceptions increased the likelihood of compliance with disinfecting surfaces by up to 28% (OR = 1.22; 95% CI: 1.13–1.31) and (OR = 1.28; 95% CI: 1.11–1.32), respectively. Having misconceptions significantly decreased the likelihood of adherence by approximately 23% (OR = 0.81; 95% CI: 0.75–0.89). Participants who perceived the community measures implemented in marketplaces, workplaces, healthcare settings, and travel places, and government decisions as effective were 1.1-1.7 times more likely to adopt disinfecting surfaces as precautions.

## Discussion

Our study analyzed the relationships among fear/anxiety levels, risk perceptions, perceived effectiveness of community-based prevention and control measures, and the public’s compliance with four self-protective behaviors (wearing facemask, hand hygiene, social distancing, and infecting surfaces). The data for the study were collected over a period of four months during the first wave of the COVID-19 pandemic in KSA, and the findings have significant implications for understanding people’s psychological and behavioral responses as well as the role of community-based measures in enhancing compliance to precautionary actions during pandemics. We interpret and discuss the findings in view of contextual factors that were operational during the first wave of the pandemic in the KSA.

Our findings demonstrate that fear levels and risk perceptions had a linear association with the intensity of the COVID-19 outbreak and closely mirrored the weekly number of new cases in the KSA. The findings indicate that the frequency of cases reported was likely to have an impact on fear/anxiety levels and risk perceptions, which significantly increased from March to June, 2020 following an increase in the number of cases in the country. These findings are somewhat contrary to the longitudinal assessment of the risk perceptions and fear levels among people in the 2003 SARS pandemic, which reported a decreasing temporal trend in the first three months of the pandemic, further dropping in the next four months.^23^ However, this contradiction can be explained in the unique context of the COVID-19 pandemic, which has affected more than 200 countries in the world in less than two to three months. Several organizations, including WHO, have launched a daily reporting system for sharing statistics about the spread of the pandemic across various regions of the world. Furthermore, the Ministry of Health within the KSA also shared daily reports on mortality and morbidity associated with the COVID-19 pandemic. This 24/7 coverage and updates through all electronic and social media channels contribute to enhancing the risk perceptions of the general public and can have negative psychological effects in the form of increasing fear and anxiety. High risk perceptions are associated with increased compliance to self-protective behaviors; however, high fear levels may lead to generalized anxiety and stress reactions. Some of the recent estimates on the prevalence of depression, anxiety, and stress among general population in KSA reached up to 25–30%.^24^

Findings show that female participants were more likely to comply with social distancing compared to male participants. These findings can be explained both in the cultural and social contexts of patriarchal societies where females are more engaged in activities limited to their homes, and those who were employed were also working from a distance during the lockdown and curfew times. There were restrictions on recreational and shopping activities during the lockdown; thus, most people complied with social distancing measures. The findings also support a three-fold increase in the likelihood of housewives complying with social distancing measures compared to those in administrative jobs. These findings somewhat align with another recent study that also demonstrated that females in the KSA are more likely to comply with self-protective measures than males.^25^ Findings demonstrated that participants aged 25–34 years are less likely to comply with social distancing compared to participants in the older group (>45 years). Saudi culture is renowned for the frequent social gatherings of male members of the community, and it was somewhat challenging to prohibit such gatherings. The local governing bodies of each region in the KSA involved law-enforcing agencies in community surveillance to ensure social distancing compliance. The Saudi government’s immediate response to deal with this public health crisis was framed under the model of “building external pressures” to promote public compliance. This was an appropriate strategy to ensure compliance with preventive measures among people from diverse cultural and social backgrounds.

Handwashing is a simple, primary preventive measure that most people can perform independently. We found that participants aged 25–34 years were less likely to adhere to hand hygiene than participants in the older group (>45 years). A possible explanation for this finding in light of the previous literature is that the individual choices made by people are in accordance with their risk perceptions.^4^ The COVID-19 infection was found to have higher mortality rates in the older age group compared to the younger age group,^17^ which might have been responsible for higher risk perceptions in older age groups. Additionally, people in older age groups are at a higher risk for comorbid medical conditions, which increases the mortality risk.^26^ These factors may increase risk perceptions, resulting in increased compliance with hand hygiene measures in older age groups compared to younger age groups. In the supplementary descriptive analysis (Table 4) of this dataset, we also found that participants’ mean risk perception scores (M = 6.71; SD = 3.14) of participants aged 25–34 years were significantly lower in comparison to participants belonging to the older group (>45 years) (M = 7.89; SD = 2.85) (*P* < 0.01). Previous literature^27^ also demonstrates that the effectiveness of control measures implemented by the governments depends, to a certain extent, on public’s response. The findings align with the literature that focuses on promoting handwashing widely throughout communities and populations well after this outbreak is contained.^28^

Despite all challenges in controlling the spread of COVID-19 infection at the community level, the current findings support that the COVID-19 prevention and control measures implemented by the Saudi government have been evaluated positively by the respondents, which significantly increased the probability of adherence to self-protective behaviors, particularly adherence to social distancing and disinfecting surfaces. The Saudi government followed the Chinese model and strictly adopted the WHO guidelines for prevention and control interventions since the outbreak of COVID-19. The rates of cases reported in the KSA during the early months of the pandemic were low compared to those reported in other parts of the world. However, the Saudi government responded promptly to the pandemic by issuing policy guidelines to govern the activities of all sectors, including health, education, business, and travel agencies. The findings demonstrated that people in the business sector were two to four times more likely to comply with wearing facemasks and disinfecting surfaces. Participants from the healthcare sector were more likely to comply with disinfecting surfaces, and people who traveled in the past two months adhered to precautionary measures of hand hygiene and disinfecting surfaces. Current study findings demonstrate that participants who complied with all four self-protective measures had higher mean scores in terms of perceived effectiveness of these community measures in marketplaces, workplaces, healthcare organizations, and places of travel. The odds ratios also demonstrate that the perceived effectiveness of government decisions greatly increased the likelihood of the public’s adoption of all four self-protective behaviors. The COVID-19 virus spread rapidly in the community through close human-to-human interactions with infected individuals. This risk is higher in marketplaces, workplaces, healthcare organizations, and places of travel as people from different families and communities visit these places. The Ministry of Health in the KSA monitors statistics and ensures strict compliance with these four precautionary measures at the community level. Compliance with self-protective behaviors at the individual level was also maintained through regular follow-ups by the government in developing policies and action guidelines for these sectors. As shown by the study findings, people who complied also highly perceived the effectiveness of these measures in helping to control the spread of the virus, as depicted by the statistics on daily new cases.

Country-based data from WHO demonstrated a significant decrease in the number of cases over the past four months in the KSA. The daily number of new cases decreased from more than 2000 to less than 500 by October 2020 in KSA.^29^ The public health experts recommend more commitment at the international level to develop worldwide awareness campaigns to prevent the spread of infection and control the deaths due to COVID-19 infection.^30^ This need for such educational campaigns is further strengthened by our study findings related to the negative relationship between misconceptions and compliance to all four self-protective behaviors.

The current study findings signify the necessity for a continuous commitment to implementing existing interventions for prevention and control, along with educational campaigns to clarify misconceptions and improve the community knowledge of the COVID-19 pandemic. The literature suggests that health promotion campaigns can include activities such as a World 2019-nCoV day or week to promote adherence to preventive actions at the national level and to provide more accurate information and address misconceptions.^30^ The Ministry of Health should also propagate the community wellness focus through media to motivate people to adjust their own behaviors at the individual level for their own well-being. These steps will help in long-term compliance with precautionary measures to prevent the expected second wave of pandemic in the region and help reduce the negative impact of the pandemic on mental health and well-being.

Previous studies and the current study demonstrate the role of functional fear as a significant predictor of self-precautionary measures during the pandemic period.^31^ The findings showed that participants who were married and had more than four children were more likely to comply with self-protective behaviors. This is possibly because of parents’ high-risk perceptions and fear regarding their children’s protection, thus resulting in their stringent adherence to precautionary measures. The findings may be explained using the extended parallel process model (EPPM), which states that risk perceptions are contingent on efficacy, defensive response, and perceived threat.^32^ According to this model, two cognitive processes are initiated when individuals are exposed to significant health risks. One of them is related to the threat it poses, and the second is related to the efficacy of engaging in the recommended behaviors (danger control). In situations where people perceive threat to be more significant and efficacy to be low, people usually act to protect themselves from fear rather than danger itself (fear control process). A recent study from Iran^33^ tested risk perceptions and adherence to protective measures under the framework of the EPPM model. According to the study findings, the fear control process led to compliance with preventive measures in more than 40% of participants and 50% were led by danger control processes.^33^ Anxiety and fear responses can motivate people to engage in precautionary behaviors^31^; however, prolonged anxiety and fear responses in masses have negative impacts on public mental health.^34^ Current findings imply that public service messages should include a component of reassurance to control the elevated anxiety response during such uncontrolled outbreaks. The COVID-19 pandemic has posed a unique public health crisis to the whole world and there are still many gaps in understanding the role of anxiety and fear factors in convincing people to comply with self-protective measures while considering their long-term mental health implications.

There are some limitations to this study, such as the implementation of online cross-sectional surveys shared over a period of four months; thus, the sample primarily comprised participants who were accessible through these platforms and agreed to respond to the survey. This resulted in a higher representation of educated participants in the sample who completed college and university level education, as well as internet users. In the context of Saudi Arabia, a large proportion of the population in labor jobs are immigrants with low levels of education. There is a lack of research on populations that are at an increased risk of getting infected and spreading the infection because of their increased exposure to less safe physical environments such as shopping malls, grocery stores, overcrowded public recreational places, and most of these people live in more congested residential areas. Second, we relied on participants’ ratings about the effectiveness of community-based measures, which have a subjectivity component; some people may have underrated the usefulness of these interventions because of their individual-level experiences, and some people may have over-rated it because of the tendency to provide socially desirable responses. Particularly, the relationship between community-based measures in the context of the COVID-19 pandemic and the public’s adherence to social distancing measures needs to be explored in other social and cultural contexts to gain greater insights into these factors. This may help in devising community interventions, which promote the voluntary adoption of precautionary measures rather than relying mainly on crisis response and governments’ management capabilities to successfully prevent the expected second wave of the pandemic.

## Conclusion

Our study findings largely endorse the intensified levels of fear and anxiety associated with the COVID-19 pandemic, showing a temporal increasing trend in fear responses and risk perceptions over four months, coinciding with an increase in case incidence. The findings demonstrate that community-based interventions should focus on programs and campaigns to engage the population segments that are currently displaying less compliance. The health education policy should not only focus on emphasizing the fatalistic consequences of the expected second wave owing to a lack of adherence to protective measures but also tackle the anxiety levels among masses. Aligning with the EPM model, adherence to self-protective behaviors can be improved by enhancing the perceived sense of control, self-efficacy, and personal responsibility for outcomes. These intervention components can be more promising in enhancing the public’s participation and cooperation to achieve the benefits of the government’s community-based measures for prevention and control during public health emergencies. Future research should focus on identifying other factors such as social environments, economic conditions, and cultural factors associated with public health behaviors to ensure long-term and voluntary adoption of self-protective behaviors, rather than relying more on forceful implementation through government interventions.

## Acknowledgements

The author acknowledges the support of professional colleagues in distribution of online survey link across different regions of KSA. We would like to acknowledge referees for their valuable comments and suggestions during review process.

## Funding

This research was funded by Scientific Research Deanship at University of Ha’il – Saudi Arabia through grant number COVID-1931.

## Competing interests

The authors declare no conflict of interest.

## Ethical approval

This study was reviewed and approved by the Research Ethics Committee at the University of Ha’il dated 08/18/2020 and approved by the university president letter-number Nr.55456/5/41.

## Authors’ contributions

Conceptualization, AAAS ans SuNH; methodology, SuNH; software, AZ; validation, AZ, FBZA and SS; formal analysis, AZ; investigation, SuNH; resources, Dr. FBZA; data curation, SuNH; writing—original draft preparation, SuNH.; writing—review and editing AAAS and FBZA; visualization, SS; supervision, SuNH; project administration, AZ; funding acquisition, AAAS.

**Table 1:** Participants’ profile and proportions of participants on adherence to self-protective behaviors (N = 400)

**Study Variables**	**Participant’s profile**	**Wearing facemask**	**Hand hygiene**	**Social distancing**	**Disinfecting surfaces**
	400 (100%)	279 (70%)	366 (91%)	353 (88%)	280 (70%)
Gender					
Female	203 (51%)	142 (70%)	187 (92%)	187 (92%)**	150 (74%)*
Male	197 (49%)	137 (70%)	179 (90%)	166 (84%)	130 (66%)
Age categories (year)					
15-24	64 (16%)	42 (66%)	61 (95%)	58 (91%)	44 (69%)
25-34	105 (26%)	71 (67%)	91 (87%)	88 (84%)	73 (69%)
35-44	138 (35%)	100 (72%)	131 (95%)	126 (91%)	97 (70%)
≥45	93 (23%)	66 (71%)	83 (89%)	81 (87%)	66 (71%)
Education					
Secondary	56 (14%)	35 (62%)	48 (86%)	47 (84%)	35 (62%)
College	93 (23%)	75 (80%)*	85 (91%)	84 (90%)	76 (81%)*
University	251 (63%)	169 (67%)	233 (93%)	222 (88%)	169 (67%)
Profession					
Student	43 (11%)	32 (74%)	43 (100%)	37 (86%)	27 (63%)
Teacher	78 (20%)	57 (73%)	69 (88%)	67 (86%)	48 (61%)
Healthcare worker	65 (16%)	45 (69%)	61 (94%)	60 (92%)	58 (89%)
Housewives	84 (21%)	47 (56%)	71 (85%)	79 (94%)	50 (60%)
Business	44 (11%)	38 (87%)**	42 (95%)*	40 (91%)	40 (91%)***
Administrative /Govt. jobs	86 (22%)	60 (70%)	80 (93%)	70 (81%)	57 (66%)
Marital Status					
Not Married	122 (30%)	77 (63%)	107 (88%)	100 (82%)	81 (66%)
Married	278 (70%)	202 (72%)*	259 (93%)	253 (91%)**	199 (71%)
Number of children					
No child	129 (32%)	86 (67%)	114 (88%)	107 (83%)	91 (70%)
1	19 (12%)	26 (53%)	44 (90%)	40 (81%)	30 (1%)
2-4	101 (26%)	75 (74%)	94 (93%)	92 (91%)	74 (73%)
> 4	121 (30%)	92 (76%)**	114 (94%)	114 (94%)**	85 (70%)
Travel in the past 2 months					
Yes	132 (33%)	97 (73%)*	126 (96%)*	118 (89%)	102 (77%)*
No	268 (67%)	182 (68%)	240 (89%)	235 (88%)	178 (66%)

*P* value significance ***P < 0.001; ** P <0.01; * P <0.05

**Table 2:** t-test results comparing participants who adhere and who do not adhere to self-protective behaviors on predictor variables (N=400)

**Predictor variables**	**Wearing facemask**		**Hand hygiene**		**Social distancing**		**Disinfecting surfaces**	
	**Yes**	**No**	**Yes**	**No**	**Yes**	**No**	**Yes**	**No**
	**M (SD)**	**M (SD)**	**M (SD)**	**M (SD)**	**M (SD)**	**M (SD)**	**M (SD)**	**M (SD)**
Fear levels	7.92***(2.85)	6.11 (2.68)	7.42 (2.91)	6.59 (3.08)	7.48** (2.91)	6.21 (2.85)	7.87*** (2.89)	6.12 (2.62)
Risk perceptions	7.37***(3.19)	5.46 (2.64)	6.90** (3.20)	5.61 (2.21)	6.97*** (3.14)	5.43 (2.96)	7.33*** (5.52)	5.52 (2.42)
Misconceptions related to COVID-19	7.61 (2.07)	9.22*** (3.29)	8.01 (2.43)	9.09 (3.98)	7.97 (2.45)	9.04** (3.45)	7.68 (2.15)	9.06*** (3.26)
Perceived effectiveness of community-based								
Measures implemented in marketplaces	8.79*** (2.02)	6.68 (2.01)	8.26** (2.16)	6.93 (2.73)	8.27** (2.19)	7.25 (2.37)	8.76*** (1.99)	6.74 (2.12)
Measures implemented in workplaces	8.33 (2.55)	7.83 (2.27)	8.25 (2.45)	7.33 (2.85)	8.28* (2.42)	7.38 (2.88)	8.36* (2.49)	7.75 (2.43)
Measures implemented in Healthcare settings	8.71 (2.10)	8.38 (1.81)	8.65 (2.01)	8.21 (2.25)	8.76** (1.92)	7.35 (2.48)	8.80** (1.96)	8.15 (2.10)
Measures implemented in Travel places	8.18 (2.52)	7.87 (2.08)	8.15 (2.8)	7.29 (2.58)	8.17* (2.41)	7.37 (2.34)	8.19 (2.53)	7.83 (2.05)
Perceived effectiveness of Govt. decisions	9.63*** (1.07)	9.01 (1.61)	9.50** (1.20)	8.75 (1.99)	9.50* (1.19)	8.95 (1.88)	9.71*** (0.95)	8.81 (1.77)

M, mean; SD, standard deviation;; *P* value significance ***P < 0.001; ** P <0.01; * P <0.05.

**Table 3:** Odd ratios (OR) to determine association of predictor variables in adherence to self-protective behaviors

**Predictor variables**	**Wearing facemask**	**Hand hygiene**	**Social distancing**	**Disinfecting surfaces**
	**OR (95% CI)**	**OR (95% CI)**	**OR (95% CI)**	**OR (95% CI)**
Gender				
Female	1.02 (0.66-1.56)^ns^	1.17 (0.58-2.37)^ns^	2.18 (1.15-4.13)^***^	1.45 (0.94-2.24)^ns^
Male	1	1	1	1
Age categories (year)				
15-24	0.78 (0.39-1.54)^ns^	2.45 (0.64-9.28)^ns^	1.43 (0.50-4.03)^ns^	0.91 (0.45-1.79)^ns^
25-34	0.85 (0.46-1.56)^ns^	0.75 (0.33-.95)**	0.76 (0.34-0.98)^**^	0.93 (0.50-1.71)^ns^
35-44	1.07 (0.61-1.92)^ns^	2.25 (0.82-6.15)ns	1.55 (0.66-3.63)^ns^	0.96 (0.54-1.72)^ns^
≥45	1	1	1	
Education				
Secondary	0.81 (.44-1.47)^ns^	0.46 (0.19-1.12)^ns^	0.68 (0.31-1.53)^ns^	0.81 (0.44-1.47)^ns^
College	2.02 (1.13-3.61)^**^	0.82 (0.364-1.95)^ns^	1.21 (0.55-2.68)^ns^	2.16 (1.20-3.91)^**^
University	1	1	1	1
Profession				
Student	1.26 (0.55-2.87)^ns^	1.11 (0.50-2.90)^ns^	1.41 (0.50-3.90)^ns^	0.85 (0.400-1.84)^ns^
Teacher	1.17 (0.59-2.32)^ns^	0.57 (0.19-1.69)^ns^	1.39 (0.60-3.21)^ns^	0.81 (0.43-1.54)^ns^
Healthcare worker	0.97 (0.48-1.96)^ns^	1.14 (0.30-4.23)^ns^	2.74 (0.94-7.93)^ns^	4.21 (1.71-10.3)^**^
Business	2.74 (1.03-7.28)^*^	1.57 (0.30-8.14)^ns^	2.28 (0.71-7.30)^ns^	5.08 (1.65-15.6)^**^
Housewife	0.55 (0.29-1.03)^ns^	0.41 (0.14-1.13)^ns^	3.61 (1.25-10.36)^*^	0.74 (0.41-1.39)^ns^
Administrative jobs	1	1	1	1
Marital Status				
Married	1.55 (0.98-2.44)*	1.91 (0.93-3.91)^ns^	2.22 (1.21-4.13)^**^	1.27 (0.80-2.01)
Not Married	1	1	1	1
Number of children				
1	0.56 (0.28-1.10)^ns^	1.15 (0.39-3.37)^ns^	0.91 (0.38-2.15)^ns^	0.65 (0.33-1.31)^ns^
2-4	1.44 (0.81-2.56)	1.76 (0.69-4.51)^ns^	2.10 (0.92 -4.79)^ns^	1.14 (0.64 -2.04)^ns^
> 4	1.58 (0.91-2.76)	2.14 (0.84-5.45)^ns^	3.34 (1.37-8.15)^**^	0.98 (0.57-1.69)^ns^
No child	1	1	1	1
Travelled in past 2 months				
Yes	1.31 (0.82-2.08)^ns^	2.45(0.99-6.07)^*^	1.18 (0.61-2.29)^ns^	1.71 (1.06-2.77)^*^
No	1	1	1	1
Fear/Anxiety Scores	1.23 (1.14-1.33)***	1.09 (0.97-1.23)ns	1.14 (1.03-1.27)**	1.22 (1.13-1.31)**
Risk Perceptions	1.21 (1.13-1.30)^***^	1.13 (1.01-1.27)^*^	1.16 (1.05-1.28)^**^	1.28 (1.11-1.32)^***^
Misconceptions	0.78 (0.72-0.85)^***^	0.86 (0.77-0.98)^*^	0.86 (0.78-0.96)^**^	0.81 (0.75-0.89)^***^
Perceived effectiveness of community-based				
Measures implemented in marketplaces				
Measures implemented in workplaces	1.08 (0.99-1.17)	1.13 (0.99-1.33)	1.13 (1.01-1.27)^*^	1.09 (1.01-1.19)^*^
Measures implemented in Healthcare settings	1.16 (1.04-1.28)^**^	1.09 (0.92-1.30)^ns^	1.31 (1.14-1.51)^***^	1.16 (1.04-1.28)^**^
Measures implemented in Travel places	1.05 (0.96-1.15)^ns^	1.14 (0.98-1.31)^ns^	1.13 (1.01-1.28)^*^	1.06 (0.97-1.16)^ns^
Perceived effectiveness of Govt. decisions	1.42 (1.19-1.68)^***^	1.33 (1.08-1.63)^**^	1.26 (1.04-1.53)^*^	1.74 (1.42-2.14)^***^

*P* value significance ***P < 0.001; ** P <0.01; * P <0.05, ns = non-significant.

**Table 4:** Comparison of mean scores on fear levels and risk perceptions across demographic variables

	**Fear Levels** **M (SD)**	**Risk perceptions** **M (SD)**
Gender		
Female	7.29 (2.93)	6.57 (3.12)
Male	7.33 (2.92)	7.01 (3.18)
Age categories (year)		
15-24	7.15 (2.91)	6.01 (3.51)
25-34	7.27 (2.91)	6.71 (3.14)
35-44	7.10 (3.01)	6.55 (3.04)
≥45	8.01 (2.66)*	7.89 (2.85)**
Education		
Secondary	7.27 (2.83)	5.62 (3.28)
College	8.69 (2.46)**	8.28 (2.87)**
University	6.90 (2.96)	6.50 (3.05)
Profession		
Student	7.18 (2.73)	5.47 (3.83)
Teacher	6.70 (2.94)	6.17 (3.21)
Healthcare worker	8.11 (2.87)	8.20 (2.71)
Business	8.91 (2.13)**	9.02 (1.98)**
Housewife	7.01 (2.83)	5.85 (3.09)
Administrative jobs	7.00 (3.15)	6.75 (2.95)
Marital status		
Married	7.29 (2.79)	7.01 (3.35)*
Not married	7.38 (2.98)	6.31 (3.05)
Number of children		
1	7.05 (2.85)	6.94 (2.63)
2-4	8.06 (2.20)	6.87 (3.22)
> 4	7.34 (3.05)	7.18 (3.06)
No child	7.41 (3.09)	6.34 (3.34)
Travelled in past 2 months		
Yes	8.08 (2.62)**	7.33 (3.14)*
No	7.00 (3.01)	6.54 (3.14)

M, mean; SD, standard deviation; *P* value significance ***P < 0.001; ** P <0.01; * P <0.05.
